# The Protonic Brain: Nanoscale pH Dynamics, Proton Wires, and Acid–Base Information Coding in Neural Tissue

**DOI:** 10.3390/ijms27020560

**Published:** 2026-01-06

**Authors:** Valentin Titus Grigorean, Catalina-Ioana Tataru, Cosmin Pantu, Felix-Mircea Brehar, Octavian Munteanu, George Pariza

**Affiliations:** 1 Faculty of General Medicine, Carol Davila University of Medicine and Pharmacy, 050474 Bucharest, Romania; 2 Department of General Surgery, Carol Davila University of Medicine and Pharmacy, 050474 Bucharest, Romania; 3 Clinical Department of Ophthalmology, Carol Davila University of Medicine and Pharmacy, 020021 Bucharest, Romania; 4 Department of Ophthalmology, Clinical Hospital for Ophthalmological Emergencies, 010464 Bucharest, Romania; 5 Department of Anatomy, Carol Davila University of Medicine and Pharmacy, 050474 Bucharest, Romania; 6 Department of Neurosurgery, Carol Davila University of Medicine and Pharmacy, 050474 Bucharest, Romania; 7 Puls Med Association, 051885 Bucharest, Romania

**Keywords:** proton microdomains, organelle nanojunctions, mitochondrial cristae energetics, hydration-structured proton conduction, cytoskeletal protonation dynamics, nanoscale bioenergetics, intracellular soft-matter physics, multiscale neural computation, phase-structured energy flow, neuroenergetic signaling

## Abstract

Emerging research indicates that neuronal activity is maintained by an architectural system of protons in a multi-scale fashion. Proton architecture is formed when organelles (such as mitochondria, endoplasmic reticulum, lysosomes, synaptic vesicles, etc.) are coupled together to produce dynamic energy domains. Techniques have been developed to visualize protons in neurons; recent advances include near-atomic structural imaging of organelle interfaces using cryo-tomography and nanoscale resolution imaging of organelle interfaces and proton tracking using ultra-fast spectroscopy. Results of these studies indicate that protons in neurons do not diffuse randomly throughout the neuron but instead exist in organized geometric configurations. The cristae of mitochondrial cells create oscillating proton micro-domains that are influenced by the curvature of the cristae, hydrogen bonding between molecules, and localized changes in dielectric properties that result in time-patterned proton signals that can be used to determine the metabolic load of the cell and the redox state of its mitochondria. These proton patterns also communicate to the rest of the cell via hydrated aligned proton-conductive pathways at the mitochon-dria-endoplasmic reticulum junctions, through acidic lipid regions, and through nano-tethered contact sites between mitochondria and other organelles, which are typically spaced approximately 10–25 nm apart. Other proton architectures exist in lysosomes, endosomes, and synaptic vesicles. In each of these organelles, the V-ATPase generates steep concentration gradients across their membranes, controlling the rate of cargo removal from the lumen of the organelle, recycling receptors from the surface of the membrane, and loading neurotransmitters into the vesicles. Recent super-resolution pH mapping has indicated that populations of synaptic vesicles contain significant heterogeneity in the amount of protons they contain, thereby influencing the amount of neurotransmitter released per vesicle, the probability of vesicle release, and the degree of post-synaptic receptor protonation. Additionally, proton gradients in each organelle interact with the cytoskeleton: the protonation status of actin and microtubules influences filament stiffness, protein–protein interactions, and organelle movement, resulting in the formation of localized spatial structures that may possess some type of computational significance. At multiple scales, it appears that neurons integrate the proton micro-domains with mechanical tension fields, dielectric nanodomains, and phase-state transitions to form distributed computing elements whose behavior is determined by the integration of energy flow, organelle geometry, and the organization of soft materials. Alterations to the proton landscape in neurons (e.g., due to alterations in cristae structure, drift in luminal pH, disruption in the hydration-structure of the cell, or imbalance in the protonation of cytoskeletal components) could disrupt the intracellular signaling network well before the onset of measurable electrical or biochemical pathologies. This article will summarize evidence indicating that proton–organelle interaction provides a previously unknown source of energetic substrate for neural computation. Using an integrated approach combining nanoscale proton energy, organelle interface geometry, cytoskeletal mechanics, and AI-based multiscale models, this article outlines current principles and unresolved questions related to the subject area as well as possible new approaches to early detection and precise intervention of pathological conditions related to altered intracellular energy flow.

## 1. Introduction—Protonics as a Missing Dimension of Neural Computation

While the motion of individual ions that follow classical electro-diffusion equations (e.g., sodium, potassium) or generate action potentials, encode synaptic probability in calcium transients, create inhibitory profiles through chloride gradients, or assist in pH and volume regulation through bicarbonate movement is typically what is referred to as neural computation, many researchers view neural computation as a collective function of all ions within a neuron [1]. While the biophysics of the motion of ions (and therefore their ability to participate in neural information processing) can be modeled using well-established biophysical theory due to the fact that the motion of ions can be described as the motion of individual particles, the proton has generally been thought of as a metabolic or homeostatic variable and not as a variable related to neural information processing. However, as researchers begin reporting results from recent studies in nanoscale imaging, single particle tracking, optogenetic pH reporters, and quantum-enabled proton sensing, there is evidence suggesting that there could be some unknown or unexplored dimensions of signaling that involve protons [2]. In addition to being smaller and lighter than other ions, protons move through biological systems via the Grotthuss mechanism, where they form a hydrogen bond network that allows protons to move at speeds greater than would be possible if they were moving randomly. The speed at which protons can move creates dynamic microdomains of acidity and alkalinity at sizes less than 1 micron and at times less than 1 ms. These properties are consistent with the size and time scales of synaptic integration and sub-cellular signaling [3].

The biophysics of proton diffusion give the nervous system properties that distinguish it from classical ionic signaling. Specifically, proton movement through hydrated layers of ions at membrane interfaces, on the surface of intracellular proteins, and in the extracellular matrix create conducting pathways that are dependent on the geometric nature and polarity of the surrounding environment [4,5]. Molecular dynamics simulations of the structure and movement of hydrated layers of ions at membrane interfaces show that the barriers to proton movement vary greatly with the composition of lipids, curvature of membranes, and the orientation of interfacial water molecules. The dielectric properties of the local environment can also lead to rapid changes in the protonation states of amino acids, allowing proteins to both sense and control proton flow [6]. The presence of aromatic residues and tightly bound water molecules can create “proton relays” that stabilize certain hydronium states for short periods of time, while charged lipid headgroups can stabilize intermediate hydronium states. Overall, these factors create a topological map of where protons interact with the architectural features of neural membranes and protein complexes in a way that is orders of magnitude more sensitive to nanometer-scale structural detail than the motion of larger ions [7].

The synaptic cleft represents the clearest example of the dependence of proton distribution on the structural features of its environment. Each synaptic vesicle contains an acidic interior with a pH of ~5.2 generated by the proton gradient across the V-ATPase that is required to load neurotransmitters into the vesicle. When the vesicle fuses with the plasma membrane, the sudden equilibration of luminal protons with the exterior environment generates a localized and transient acidification event [8]. Recent studies have shown that the use of ultra-fast fluorescent proton reporters demonstrates that these acidification events can produce acidification transients with amplitudes of tens of pH units and timescales of milliseconds. These acidification transients alter the kinetic properties of postsynaptic receptors; specifically, the likelihood of opening of NMDA receptors decreases substantially with even small acidifications and modifies both Mg^2+^ block kinetics and Ca^2+^ influx profiles [9]. Acid-sensing ion channels rapidly respond to these acidification transients by producing currents that either amplify or suppress subthreshold fluctuations in membrane potential based on the local buffering capacity. The TRP family of channels, including TRPV1 and TRPV4, undergo pH-dependent conformational changes that modify their conductance states, thereby adding another layer of complexity to synaptic transmission [10]. Taken together, these data demonstrate that synapses convey a composite signal that includes the concentration of neurotransmitter, membrane potential, and structured proton dynamics, each providing distinct temporal and spatial information [11].

Proton distributions in the cytosol are determined by the interaction of metabolic flux, organelle shape, and the activities of exchangers, cotransporters, and carbonic anhydrases. Mitochondria generate proton electrochemical gradients that are required for oxidative phosphorylation; minor alterations in mitochondrial inner membrane potential or the shape of cristae can alter these gradients and impact ATP production and redox state [12]. Proton leaks through uncoupling pathways can decrease the efficiency of energy conversion and lower the excitability threshold of neurons when under metabolic stress. Endosomes, lysosomes, and the Golgi apparatus contain different pH values that are essential for trafficking and enzymatic processing; disruptions in these pH gradients can affect synaptic turnover and proteostasis [13]. Intracellular pH gradients within dendritic spines affect the buffering of calcium, the rate of dephosphorylation, and the conformations of proteins that regulate actin organization in dendritic spines. Spine morphology, particularly the resistance of the neck compartment, influences the interaction of proton diffusion so that localized acid-base microdomains can form in response to intense activity and affect plasticity thresholds and meta-plastic states [14].

There is also a higher-order level of proton-dependent behavior displayed by the extracellular domain. Physiological oscillations can produce activity-dependent changes in extracellular pH that typically include brief alkalinizations that increase neuronal excitability. Prior to seizure onset, there is a transient increase in extracellular pH that increases NMDA receptor currents and decreases inhibition through changes in bicarbonate conductance [15]. Conversely, spreading depressions, ischemia, and hypoxia all produce large extracellular acidifications that inhibit synaptic transmission, modify the activation curves of voltage-gated channels, and slow down receptor kinetics. Astrocytes are responsible for regulating the dynamics of pH in the extracellular space through Na+/H+ exchangers, bicarbonate transporters, lactate-proton cotransporters, and the spatial buffering of proton loads throughout the neuropil. Due to the high mobility of protons in structured extracellular water, these dynamics can travel as “acid-base waves,” coordinating regions of the brain during transitions between network states [16].

Collectively, these results demonstrate that proton distributions are not simply indicators of cellular metabolism but are actively engaged in the integration of signals, modulation of excitability, regulation of enzymes, and switching between states among populations of neurons. Additionally, the unique physical properties of protons provide modes of communication and regulation that are fundamentally different from diffusion-driven ionic processes [17]. As new methods for measuring proton behavior become available—such as genetically encoded ultra-fast pH fluorophores, nanoscale fiber optic proton sensors, quantum NV-center proton mapping, and atomistic simulations that model real-time hydrogen bond kinetics—we are beginning to understand patterns of proton behavior that were previously inaccessible. These advancements offer the possibility to consider the roles of acid-base dynamics as a separate, computational layer within neural tissue [18].

This review aims to bring together recent primary research from molecular biophysics, neurochemistry, electrophysiology, metabolism, and nanoscale imaging to provide a conceptual framework for understanding proton-based signaling within the brain. The ultimate goal of this review is to describe how proton gradients are formed, how they are created and altered by membranes and protein complexes, how they affect the physiological functions of neurons and glial cells, and how their disruption contributes to disease. This review draws together theoretical models, experimental measurements, and structural simulations from a variety of fields to present a comprehensive overview of the current knowledge of protonic processes and how they contribute to information processing. The next several sections will address the nanoscale proton architecture, the variety of proton-sensitive ion channels and transporters, the general principles of proton-based coding, proton circuits that utilize metabolic and organelle components, and the possible contributions of proton dysfunction to a variety of neurological disorders, with the hope of stimulating further explicit investigation of protonics as an additional dimension of neural computation that was possibly overlooked.

## 2. Quantized Ionic Microphysics and Multiscale Energy Transduction in Living Neural Tissue

There must be a full understanding of quantum mechanics, nanoscale biology, the organization of soft materials and the electrochemistry of organelles to understand how protons participate in neural information processing. At the interfaces of biological systems, protons exhibit a unique characteristic relative to all other ions; instead of behaving as individual discrete particles, protons behave as delocalized defects in charge because of the formation of hydrogen bonds in the networks of water and amphipathic matrices that reorganize structurally at different velocities and with different coherence properties relative to classical diffusion [19]. A description of the physical principles involved in the movement of protons in neural tissue will be provided in this section and the mechanisms of proton movement will be described at the scales of femtoseconds, nanometers, and single interfaces. The intent is to describe the limitations and opportunities of proton transport as they exist in membranes, proteins, organelles, and computational substrates composed of nanofilm water; each of these constitutes a distinct computational substrate as defined by the electrostatics, topological properties, dielectric geometry, and quantum tunneling physics of the system.

### 2.1. Quantum Proton Mobility in Confined Biological Environments

Protons do not diffuse through living cells as hydrated H^+^ ions; instead, they travel through coordinated reorganization of the hydrogen bonding arrangements that convey excess charge between networks of water molecules or polar residues. Events that involve the very fast reorganization of structural arrangements and partial delocalization of charge through Grotthuss-like relay mechanisms occur at sub-picosecond time scales. In the constrained geometrical context of neural systems—e.g., the synaptic cleft, perimembrane hydration layer, vestibule of an ion channel, interfaces of adjacent organelles etc.—the hydrogen bonding connectivity of the water molecules is greatly anisotropic and provides directionality to the movement of the proton [20]. Recent ultrafast, two-dimensional infrared spectroscopy experiments suggest that the confinement of the proton reduces the number of possible hydrogen bonded configurations that can be accessed by the proton and therefore increases the probability that the proton will travel through “quantum channels” formed by chains of water molecules whose orientation is defined by the local surface topology [21,22].

Quantum tunneling provides additional mobility of the proton through narrow constrictions where hydrogen bonding distances are periodically reduced by thermal or electrical fluctuations. Ab initio molecular dynamics simulations that are coupled to path integral methods show that the tunneling probabilities of protons are enhanced in proximity to carbonyl-rich surfaces, membrane headgroups, and protein interior cavities in which transient alignment of water chains can occur [23]. Tunneling does not require a complete reorganization of the network of water molecules in the vicinity of the proton and therefore introduces non-classical modes of conduction in which proton flux can be responsive to high-frequency electrical activity or rapid conformational transitions [24].

Dielectric inhomogeneities at the local scale modify the proton energetics. For example, near lipid bilayers, water molecules form semi-ordered configurations that restrict the rotational freedoms of the water molecules and create conduction corridors for protons that have altered activation energies [25]. Similarly, dielectric microenvironments exist at the interfaces of the cytoskeleton, where the density of acidic residues is sufficient to create gradients in the ordering of the hydration shells [26]. These gradients affect the effective mass of the proton and the curvature of its potential energy landscape and therefore relate the geometry of the neural system to the quantum mobility of the proton [27].

These studies collectively demonstrate that confined quantum proton dynamics provide a physically reasonable mechanism for ultrafast signal components that cannot be explained by either ions or classical second messengers. However, the computational significance of these proton relays is currently under active investigation and it appears that neurons contain several architectures that support such proton relays [28].

### 2.2. Membrane, Protein, and Water Nanostructures as Proton-Conduction Landscapes

While the quantum behavior describes the proton motion at the smallest scales, the architectural structure of neural tissue defines the paths along which proton charge defects can propagate. The plasma membrane represents the primary interface at which lipid head groups and their hydration layers, as well as embedded proteins, define a heterogeneous conduction topology [29]. Different phospholipid species have different pKa values, dipole moments of the head groups, and hydrogen bonding patterns that define a mosaic of proton-buffering capacities that are spatially distributed over scales of nanometers. Spatial gradients in lipid composition from sphingomyelin-rich regions to PIP_2_-rich micro-domains define spatially structured surface pH profiles that affect proton retention, dissipation, and lateral conduction [30,31].

Membrane proteins embedded in the plasma membrane also define structured pathways for proton conduction. Proton channels, pumps, and proton-coupled transporters utilize chains of acidic/basic residues, bound water molecules, and dynamically gated hydrophobic gates that open transiently to allow proton translocation. These pathways are not simply conduits but tunable energy landscapes [32]. Individual point protonation events affect the conformational ensemble of the helices of the channel, affect the ordering of the hydration shell within the vestibules, and affect the local electrostatic gradients. Consequently, proton flux is regulated through cooperative transitions between protein backbone states and fluctuating connectivity of the water chain [33].

Cytosolic proteins have internal hydration networks that can conduct small numbers of protons. Cavities defined by beta-barrel and/or alpha-helix bundles and/or intrinsic disorder create confined aqueous spaces in which the hydrogen bonding networks of the cavity fluctuate under mechanical and/or electrostatic stress [34]. Protonation of certain residues in these cavities causes conformational micro-adjustments in the protein backbone that can propagate through the protein, thereby defining the possibility of encoding information in the trajectory of the proton’s passage through the protein in terms of residue charge patterns, protein backbone dynamics and/or protein–protein interactions [35]. Therefore, the existence of these mechanisms supports the possibility of proton-induced conformational computing [36].

Quasi-2D proton conduction can also occur in nanoscale water films that surround membranes, cytoskeletal structures and surfaces of organelles. Dielectric constants and hydrogen bonding lifetimes of the hydration films are different from those of bulk water and therefore define proton pathways and localized pH microdomains in space. Dendritic shafts, axonal internodes, and presynaptic terminals contain hydration films that constitute structural pathways that direct proton flux through the neural tissue independently of classical ion diffusion [37]. Changes in the thickness of the hydration films, the curvature of lipids and/or the anchoring of cytoskeletons can locally change the proton conduction velocity and stability of proton pathways, thereby defining the proton signaling topology of the neural tissue based on mechanical and/or metabolic states [38].

Therefore, the membrane-protein-water ensembles described above define dynamic landscapes that specify the location, mechanism, and rate at which proton charge defects move and connect the microstructural organization of neural tissue to the computational repertoire of proton-based signaling [39].

### 2.3. Coherent Proton Pathways Across Organelles and Subcellular Interfaces

The proton dynamics of neurons are defined by both the membranes and the hydration films and by the networks of organelles that define proton gradients, patterned pH domains, and organized proton transfer interfaces. Mitochondria generate large proton motive forces across their inner membranes and can define proton gradients that exceed 180 mV and proton microdomains at the contact points of the outer membrane, affecting the hydration networks of the surrounding cytoplasm and the acidity of the hydration film surrounding the mitochondria [4,40].

At the interfaces of the ER and mitochondria, the access of protons to the interface is controlled by the nanoscale structures that tether the membranes together and the distance between the membranes (typically 10–25 nm). This distance is sufficiently narrow to allow for structural water layers to persist, yet sufficiently large to permit the hydrogen bonding networks to fluctuate [41]. It appears that proton capture and transfer at these interfaces depend on microchannels associated with VDAC, patches of acidic lipids and clusters of charges generated by the tethering complexes [42,43]. Therefore, the interfaces of the mitochondria and the ER constitute proton exchange hubs whose protonation states change in response to local electrical and/or metabolic changes, thereby changing the proton flux between compartments [42].

Synaptic vesicles and lysosomes are compartments that define proton-rich environments internally due to V-ATPase activity that generates large proton fluxes into these compartments. The proton-rich environment of the compartment interiors creates sharp proton gradients at the surface of the compartments [44]. Important high-speed pH nanosensors have shown that proton transients are released during exocytosis and these transients may provide local modulation of the ordering of the water in the synaptic cleft, of the dynamics of the buffering of the extracellular space, and/or of the protonation states of receptors; they therefore add another physical dimension to the process of neurotransmission [45].

The proton-rich microenvironment of peroxisomes, late endosomes, and autophagolysosomal compartments influences the topology of the hydrogen bonding arrangements of the water molecules in the hydration layers surrounding these compartments, and may interact with redox fluctuations to modulate proton-coupled electron transfer reactions and/or change the physicochemical state of the local condensates, interface viscosities, and/or protein conformational ensembles [46].

A higher level of organization exists when proton-dependent communication between organelles occurs. The proton flux between mitochondria and lysosomes appears to coordinate the metabolic demand of the cell with the degradation capacity of the lysosomes. The proton transients that exist at the ER-lysosome junctions appear to regulate the luminal acidity of the lysosomes during calcium release cycles, thereby defining the relationship between proton dynamics and ionic signaling [47]. Therefore, the proton-dependent communication between organelles defines nonlinear feedback loops in which the changes in proton activity affect the structural, metabolic, and mechanical states of the cell [48].

**Figure 1:**
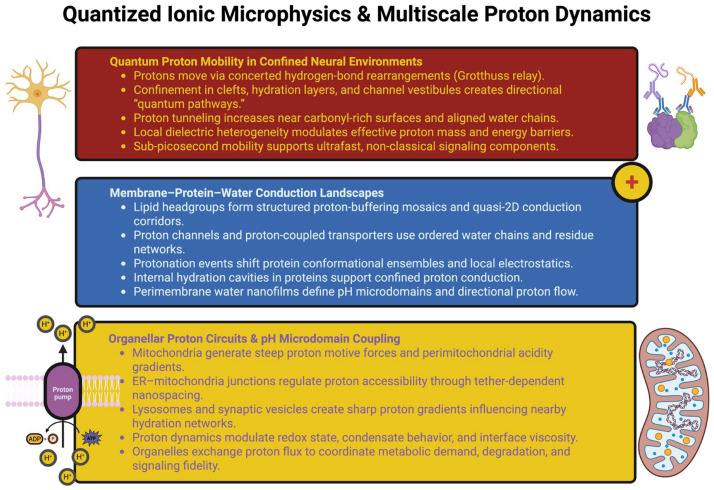
Demonstration of how proton behavior within neural tissue is created by the interplay of three physically connected layers.

Therefore, the proton circuitry between organelles defines a spatially resolved, multi-compartmental proton landscape that relates the metabolism, structural state, and local signaling topology of the neuron. Instead of being passive byproducts of cellular energetic processes, proton gradients at organelle interfaces appear to be active components of intracellular computation, influencing conformational dynamics, membrane tension, electrochemical flux and/or the nanoscale architecture of hydration in the neuron [49,50]. Figure 1 summarizes how proton behavior in neural tissue emerges from the interplay of quantum mobility, membrane–protein–water conduction architectures, and organelle-driven pH microdomains.

## 3. Proton-Dependent Modulation of Neural Signaling and Molecular State Transitions

Protons, acting as a physical variable, modulate the neural computational process through several different mechanisms, far beyond those that could be anticipated from an acid-base chemistry perspective alone. Due to the fact that the protonation of a site will change the electrostatics, hydrogen bonding, and free energy of conformational transitions with high spatial resolution, proton flux represents a variable that enables the restructuring of neural signaling over a temporal range that extends from femtosecond to second timescales [51]. With the ability of protons to reorganize active site chemistry, restructure hydration networks, and affect the dielectric constants of biological membranes and protein domains, proton dynamics represents a critical and previously under-appreciated factor that determines synaptic, dendritic, and axonal computation [39,52]. This section will discuss how proton availability modulates neural signaling through alterations in conformational microstates, enzymatic kinetic parameters, vesicle biophysics, and integrated microcircuit behavior across several sub-cellular domains.

### 3.1. Proton-Controlled Conformational Microstates in Ion Channels and Transporters

Ion channel behavior is modulated by proton activity through electrostatic nano-scale modifications that shift the ensemble of possible conformational states of the gating domain. Certain acidic residues located in clusters near the pore entrance of voltage-gated sodium, potassium, and calcium channels function as proton-sensitive charge regulators [53]. Protonation of these acidic residues alters the hydration shell geometry and changes the balance between the electric field that drives voltage sensor movement and the mechanical resistance imposed by adjacent lipid head groups. The slight distortion of this electric field shifts the potential energy surface governing S4 helix movement and alters the probability of intermediate gating configurations and the activation threshold with remarkable temporal resolution [54].

Ion-gated receptors similarly exhibit significant proton-mediated modulation. Protonation of the glutamate binding pocket in AMPA and kainate receptors influences the hydrogen-bonding networks between the clam-shell domains and modulates the dwell-time distribution of the ligand and the trajectory of receptor desensitization. In NMDA receptors, proton binding to allosteric inhibitory sites reduces pore hydration and stabilizes the closed configuration of the M3 helix bundle, enhancing both the calcium-permeability and the open-probability of the pore [55]. These events occur at a temporal scale that is many orders of magnitude faster than the rate of protein turnover or trafficking, thus enabling proton dynamics to modulate excitatory synaptic gain in real time.

Proton-coupled transporters appear to use proton binding as an internal timing mechanism. The binding of a single proton to a deeply buried acidic residue of the transporter typically reorganizes the inner hydration networks of the transporter and decreases the dielectric screening and stabilizes the transient conformation(s) that favor substrate translocation. Proton-driven conformational “ratcheting” has been observed in amino acid, monocarboxylate, and neurotransmitter transporters, thus providing a mechanism whereby small changes in pH can modulate nutrient uptake and neurotransmitter recycling [56,57]. In vesicular GABA and glutamate transporters, the luminal proton gradient determines the stoichiometry and energetics of neurotransmitter loading and therefore modulates the composition of synaptic release and quantal variability. In terms of physics, protonation acts as a low-energy, high-speed switch that modifies the protein energy landscape with high spatial specificity because proton binding requires very little structural rearrangement. Thus, proton binding events represent one of the fastest physicochemical means by which neurons can modify their electrical and biochemical behavior [58].

### 3.2. Proton-Regulated Enzymatic Kinetics, Energy Barriers, and Proteome Dynamics

Proton availability modulates enzymatic function throughout the neuron through the alteration of catalytic geometries, active-site dynamics, and reaction branching probabilities. Many of the catalytic residues involved in phosphorylation/dephosphorylation, methylation/acetylation, and redox chemistry depend upon protonation states to modulate nucleophilicity, electrophilicity, and/or transition-state stabilization. Modest changes in proton availability modulate the orientation of catalytic aspartate and histidine residues in protein kinases, thus altering the hydrogen-bonding networks that stabilize the transition-state [59]. This modulates the phosphorylation-rates and the oscillatory kinetics of the signaling-module, such as MAPK, CaMKII, and PKA. Proton-mediated changes in activation-loop protonation modulate the stability of active and inactive conformations and introduce pH-dependence into the stability of these conformations to enable precise spatiotemporal-tuning of phosphorylation cascades. Redox enzymes show similar dependencies on proton availability. Redox reactions involving proton-coupled electron transfer (PCET) require the simultaneous motion of electrons and protons through structured hydrogen bond networks [60,61]. Modest changes in protonation can alter these networks, thus modulating reaction barriers and changing the spatial distribution of reactive oxygen species (ROS) produced at basal or high-activity levels. Given the strong interaction of ROS with cysteine, methionine, and aromatic residues in signaling proteins, proton-tuned redox micro-domains can broadly modulate neural function, particularly during synaptic bursts or metabolic transitions [62].

Proton availability also modulates the regulation of the cytoskeleton. In actin filaments, protonation of acidic residues near polymerization interfaces can alter the stiffness of the monomer–monomer connections, thus modulating the persistence-length of the filaments and the response of dendritic spines to synaptic input. Moreover, there exist proton-sensitive, actin-binding proteins, including ADF/cofilin and profilin, whose pH-dependent binding affinities regulate filament turnover during plasticity and modulate the mechanical compliance of spines and dendrites during activity-dependent remodeling [63]. Liquid–liquid phase-separated protein condensates undergo proton-dependent transitions among liquid, viscoelastic, and gel-like phases. Protonation of low-complexity domains modulate sticker–spacer-interactions, reorganize hydration networks, and modulate the surface tension at the interface of the condensate [64]. These changes modulate the permeability of the condensate to signaling enzymes, modulate ribosomal access to local translation, and regulate the sequestration of transcriptional regulators in the nucleus. Proton-driven phase transitions provide a physical regulator of biochemical compartmentalization and shape the micro-environmental geometry in which signaling reactions take place [65].

Due to the effect of protonation on enzymatic kinetics and protein assembly, proton-flux provides a direct contribution to the determination of reaction rates, conformational equilibria, and the dynamic proteome architecture that supports synaptic and dendritic stability [66].

### 3.3. Proton Shaping of Synaptic Microphysiology, Vesicle Cycling, and Signal Integration

Synaptic function is highly sensitive to transient proton dynamics generated in the synaptic cleft by neurotransmission, vesicle cycling, and metabolic processes. High-frequency firing generates proton pulses in the synaptic cleft that modulate receptor behavior by modifying ligand-binding kinetics, gating energetics, and pore hydration states. The electrostatic environment in which neurotransmitter molecules diffuse in the synaptic cleft is modulated by changes in cleft proton activity which therefore modulate the receptor occupancy patterns [67,68].

Synaptic vesicles act as a concentrated reservoir of luminal protons generated by V-ATPase activity. The steep proton gradient across the vesicle membrane is necessary for efficient neurotransmitter loading; however, it also serves as a physical signal during exocytosis. Upon vesicle fusion, the rapid mixing of luminal protons with cleft water generates transient proton microbursts that reorganize the hydration shell geometry around postsynaptic receptors, potentially modulating channel open probability and short-term synaptic plasticity [69]. Likewise, proton-driven changes in vesicle luminal conditions modulate the loading efficiency and contribute to heterogeneity in quantal size and synaptic variability. At the presynaptic terminal, proton availability modulates the mechanical energy landscape of fusion. Protonation of acidic headgroups modulates the curvature stresses in the membrane, thus modulating the feasibility of hemi-fusion stalk formation and the energy barrier for fusion pore expansion. Proton activity also modulates the electrostatic interactions within the SNARE complex, thus modulating the likelihood of successful membrane merger during synchronous and asynchronous release modes [70]. At the postsynaptic terminal, proton-dependent modulations of membrane charge distribution modulate the organization of receptor clusters and scaffold dynamics. The local proton availability modulates the organization of nanodomains containing AMPA, NMDA, and neuromodulatory receptors, and modulates their lateral mobility and alignment with presynaptic release sites. Protonation also regulates dendritic calcium micro-domains by modulating the interaction between buffering molecules and cytosolic water structure, thus modulating the integration window and dendritic excitability [71].

Collectively, these mechanisms illustrate that proton flux can provide tunable, nanoscale regulation of synaptic computation and modulate vesicle behavior, receptor response, and intracellular integration without altering the number of proteins present in the synapse or network architecture. Proton dynamics provide an additional degree of freedom in regulating synaptic and circuit-level computation by serving as a physical layer that interacts with classical neurotransmission [72]. Table 1 aims to distill the core mechanisms through which proton dynamics modulate neuronal computation.

## 4. Proton-Encoded Computation Across Neural Microcircuits: Integration, Routing, and Multimodal Coupling

A number of researchers have studied the ways in which proton migration affects the physical micro-environment in which neurons communicate. These studies indicate that the proton migration process has the potential to affect energetic barriers to conformational transition, hydration topologies, local electrostatics, and the dielectric structure of the membrane and mechanically coupled state variables, each of which represents a micro-domain property that influences molecular kinetics, meso-scale coupling, and reaction accessibility [7,78]. Since protons can influence hydrogen bonding networks, unlike classical ions, protons can also alter the micro-domain properties that influence molecular kinetics, meso-scale coupling, and reaction accessibility. Data obtained from experiments and simulations demonstrate that protons can alter micro-domain properties, supporting the hypothesis that protons contribute to protonic information processing. By definition, protonic information processing is the ability of a particular proton micro-state (a particular protonation pattern in a given region of the cell, the local proton gradient, the hydrogen bonding connections in a particular area, etc.) to (i) influence the probability of a molecule or mesoscopic component being in one state versus another, (ii) perform conditionally gated, non-linear thresholding, and (iii) sustain sufficient temporal duration to affect subsequent state transitions as a transient material constraint. In this article, “computation” refers to the rule-based updates of states derived from the physical micro-states of cells and not metaphorical reasoning; some of the proposed computational interpretations are provided with caveats for testing in the future [79]. Subsequent subsections will explore how proton signals enable routing, non-linear gate operations and hybrid computation models through interaction with ionic, mechanical, and phase-state processes.

### 4.1. Proton Routing Networks and Spatial Logic in Dendritic and Axonal Continuums

Due to the hydration shell of the membrane, the membrane curvature, and the spatial arrangement of protonatable sites, proton migration is restricted in dendrites and axons. Due to the fact that proton transfer in the hydrogen bonded network of interfacial water occurs via cooperative reorganization of hydrogen bonding networks and not simply through the diffusion of classical ions, proton transfer is very sensitive to the nano-scale architecture of membranes, cytoskeleton filaments, and organellar surfaces [7,80]. At the nano-metric scale of water layers, hydrogen bonding facilitates anisotropic conduction, and thus two-dimensional hydration films can serve as dynamic wave guides. Membrane curvature and/or clustering of lipids with large dipole moments can define the direction of proton hopping and establish physically driven conduction paths [81]. Regions of proteins and/or complex structures can disrupt the continuity of hydrogen bonding and divert flow to alternative paths. Collectively, these mechanisms suggest that proton conduction can generate geometry-dependent routing networks that cannot be described as having a fixed channel topology. Repeated localized stimulation can result in longer lasting hydration rearrangement, and therefore localized activity can develop hydrogen bonding connectivity and form corridors of relatively high conduction that endure after the original stimulation [82].

Although both experimental and simulation data exist that demonstrate that stimulation can result in hydration remodeling, whether the persistence of such hydration rearrangements constitutes a memory-like trace is still a hypothesis that needs to be tested directly. A memory-like trace would represent a material constraint influencing subsequent propagation but would not represent a memory system by itself [83,84]. Additionally, proton routing does not require a single route: multiple concurrent conduction pathways with distinct hopping kinetic parameters can coexist in the same dendritic section and therefore can produce time and dispersion patterns analogous to interference, representing analog features of recent neural and metabolic histories. Routing nodes can be generated by dielectric heterogeneity resulting from differences in lipid packing, the orientation of membrane dipoles or the fixation of cytoskeleton and determine divergence and convergence behaviors [85]. The ratio of branching is most likely a function of local buffering capacities and pKa distributions of microdomains, establishing a relationship between chemistry and routing [86]. Therefore, the above findings support a conceptual framework in which the dendritic and axonal micro-geometry and hydrogen bonding connectivity provide a type of “spatial logic,” i.e., state-dependent routing rules that determine which downstream micro-domains will be subject to proton-mediated modulation [87].

In summary, geometry and hydration topology can affect proton propagation and branching. However, whether the persistence of hydration offers a plausible mechanism for history-dependent routing remains to be established.

### 4.2. Proton-Dependent Nonlinear Gating, Thresholding, and Biophysical Logic Transitions

Protons can induce non-linear transformations in neural signaling by modifying the energy landscape of conformational transitions of channels, receptors, transporters, and scaffold proteins. Modification of the local electric field, hydrogen bonding organization, and nearby helix dipoles resulting from protonation of a strategically positioned acidic or histidine residue can modify the activation barrier, similar to classical allosteric regulation [88,89]. Functionally, protonation is conditional gating: the functional outcome of the same input can vary as a function of proton activity within micro-domains. Clustered proton binding to sets of residues can produce strong non-linearities, including bistability (high/low activity states) and hysteresis (dependence on exposure history rather than instantaneous pH). When collective proton binding leads to strong non-linearity, continuous pH variation can be transformed to discrete-like conformational outputs, and therefore a mechanistic analog-to-digital transformation can be incorporated into the membrane [90].

**Figure 2:**
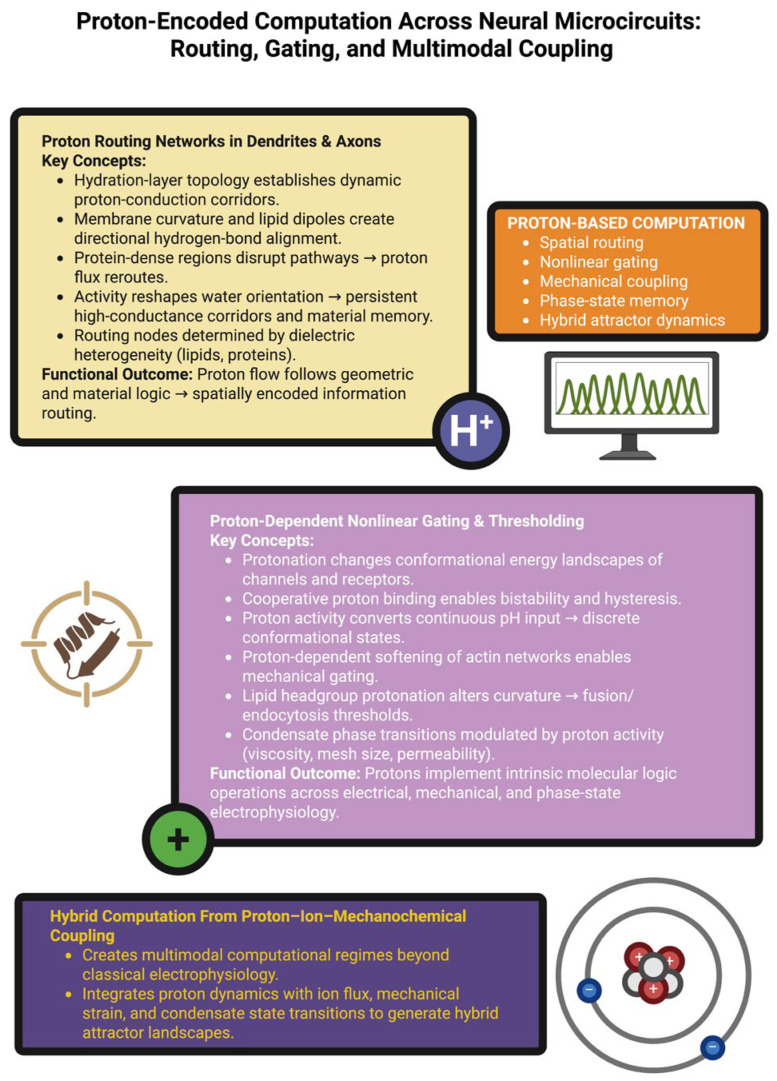
Illustration of how proton dynamics introduce a distinct computational layer into neural microcircuits by shaping routing pathways, nonlinear gating behavior, and hybrid mechanochemical–electrochemical processing modes.

This should not be interpreted as digital computation in its strictest sense, but rather as the discretization of states arising from the non-linear proton–structure interaction. Protonation also modifies mechanical and phase state variables [91]. Protonation of residues associated with actin can change the stiffness and mechanical continuity of filaments, and thus provide proton-dependent mechanical gates for the transmission of forces across spines and dendritic junctions. Proton-induced changes in the charge of lipid headgroups can change the curvature stress of membranes and therefore the barrier to fusion/endocytosis [92]. Furthermore, proton fluctuations can reorganize the internal structure of condensates and therefore influence viscosity, reaction rates, and protein sequestration, whereas the composition of condensates can influence cytoplasmic buffering and charge distribution. Collectively, these mechanisms provide a biophysical basis for proton-dependent threshold regulation and switch-like transitions across chemical, mechanical, and phase domains, and therefore can affect context-dependent signaling and reaction environments [93]. Figure 2 aims to highlight how proton dynamics shape information processing beyond classical electrophysiology.

In summary, protonation can create bistability, hysteresis, and thresholding by changing the energy, mechanical, and phase landscapes. There is a considerable body of literature supporting the effects of protonation on signaling, and the manifestation of these effects as computational primitives is reasonable and explicitly testable.

### 4.3. Hybrid Computational Modes Emerging from Proton–Ion–Mechanochemical Coupling

There appear to be several significant scenarios in which proton dynamics interact with electrical activity, mechanical strain, and phase organization. Proton–ion interactions can modulate ionic conductivity through proton-dependent changes in electrostatic fields, hydration structure, and dielectric microenvironments. For instance, protonation near sodium or calcium channels can change the organization of hydration shells and thus influence the occupation of ions, the kinetics of channel opening/closing, and peak currents [94]. Similarly, proton “microbursts” in the synaptic cleft can temporarily modify the extracellular micro-gradients and the local voltage boundary conditions and thus influence the ion diffusion fields during high-frequency activity. Thus, there exists substantial evidence for a hybrid signaling paradigm, in which the propagation and backpropagation characteristics of action potentials can be modified by the protonation history in micro-domains of the membrane [95]. Similar to this, mechanical–proton coupling introduces state dependence: deformation of the membrane can compress hydrogen-bonded networks and thus increase the hopping efficiency, and protonation of cytoskeletal interfaces can change the stiffness and mechanical continuity [96]. Thus, there is bidirectional coupling: mechanical events (e.g., expansion of spines, deformation of dendrites, tension in axons) can modify proton routing, and proton micro-states can modify mechanical response properties [97]. Therefore, the history of mechanical events can be encoded into the patterns of proton propagation through persistent material constraints, but the magnitude and timescale of this encoding need to be tested experimentally. Lastly, phase-state–proton coupling adds to these dynamics. Fluctuations in proton concentration can reorganize the internal structure of condensates, and therefore can influence viscosity, reaction rates and protein sequestration, whereas the composition of condensates can influence cytoplasmic buffering and charge distribution [98]. Collectively, these mechanisms suggest the possibility of hybrid regimes exhibiting relatively stable proton landscapes, persistent mechanical configurations, and long-duration phase architectures reflecting recent activity. Multistability and coupled state persistence have been observed in general terms in cellular systems, but the interpretation of such regimes as a memory substrate independent of synaptic weight modification [99] is speculative and requires direct testing. More conservatively, hybrid proton–ion–mechanochemical coupling is expected to increase the range of accessible dynamical regimes in neural circuits over time scales and to allow computations dependent on multi-physical contexts rather than solely on electrical signaling [100].

## 5. Multiscale Proton–Organelle Coupling: Structured Energy Flow, Nanojunction Signaling, and Computational Microdomains

Protons play several roles in the neuronal cell and perform numerous functions that go well beyond simply providing a means to maintain the acid–base balance of the cell. The protons create an energy profile within the cell which includes aspects of the function of the mitochondria, the geometry of the organelles, the topology of the hydration layer, the dielectric state of the membrane’s surface, and the mechanical and electrical states of the cytoplasm [101]. The organization of the hydration layer and the curvature confinement of protons and proteins at the interfaces between organelles and membranes at the nanometer scale creates micro-domains in which the pH varies and the kinetic relaxation time and energy threshold varies [102,103]. The proton transfer within these micro-domains is governed by the hydrogen bonded network of water molecules along the membrane surfaces and at the interfaces between organelles and membranes. Also, the affinity, buffering capacity, and local dielectric environment of the proton govern the proton pathway, rather than the diffusion coefficient of the proton [86]. The aforementioned physical principles demonstrate that organized proton micro-states can represent the integrating variables relating the metabolic states, redox transitions, membrane curvatures, mechanical stresses, and vesicle recycling and create a “common physico-chemical language” for the representation of mesoscopic transition states [104]. The next section will discuss the detection, transmission, and decoding of proton signals in mitochondrial, endolysosomal, secretory vesicle, and cytoskeletal systems across scales and the differentiation between physical mechanisms and computational models that represent signals.

### 5.1. Mitochondrial Proton Motive Force, Cristae Micro-Compartmentalization, and Energy-Dependent Proton Signatures Across Neuroarchitecture

One of the most heavily regulated proton gradients is the proton motive force (PMF) of the mitochondria’s inner membrane in neurons. Cryo-Electron Tomography (CET) coupled with fluorescent pH-reporting methods demonstrated that the curvature-associated proton micro-compartment (sub-cristae proton wells) of the mitochondria have a higher proton concentration than the surrounding inter-membrane space [105]. It is believed that the geometric asymmetry of the sub-cristae proton wells at the nanometer-scale confines protons within the sub-cristae wells and limits the lateral diffusion of protons in sub-cristae with radii > 20 nm, as well as produces pH plateaus by modifying the topological structure of the hydrogen bonding of the hydration layer. Changes in the shape of the cristae ridges during synaptic bursts can reorganize the sub-cristae proton wells and generate synchronized proton oscillations consistent with the dynamic turnover of the respiratory chain [106,107].

Proton loading/unloading in sub-cristae compartments can affect the ATP production rate by affecting the effective rotor torque per unit of ATP synthase due to proton crowding near the F0 entrance, and produce micro-domain heterogeneity in ATP production. Therefore, the workload can be represented not only by global redox variables but by phase-shifted pH-profiles transmitted through the cytoplasm using nano-junctions created at the mitochondria-ER contact sites (MERCs) by defining “energy signature” as the operational definition of the reproducible mapping between proton micro-domains and measurable outcomes (e.g., ATP yield heterogeneity, Ca^2+^ regulation, metabolite exchange kinetics) instead of an actual symbolic code [108].

Nano-junctions (the MERCs) are proton-sensitive interfaces found between closely apposed membranes (distance ~10–25 nm) that allow the hydration layers at the interfaces to couple and rapidly adjust the micro-environment of nearby proteins (e.g., IP_3_ receptors, SERCA pumps, MCU-MICU channels). This interface can form an electro-steric feedback loop: the proton flow from the mitochondria can modulate the permeability of ER Ca^2+^, and the ER Ca^2+^ flow can modulate the mitochondrial membrane potential and therefore rearrange the proton confinement [109,110]. Furthermore, pH-sensitive lipid nanodomains, particularly those containing cardiolipin and having altered local pKa values on the outer mitochondrial membrane, can create dielectric gradients that can modulate the conductance of VDAC and regulate the exchange of metabolites and redox substrates. Thus, the proton dynamics of mitochondria can provide a method for transforming metabolic load and activity state into organized energetic micro-domains that can serve as state variables for controlling subsequent decision-making processes and signaling [39,111].

### 5.2. Lysosomal, Endosomal, and Vesicular Proton Architectures Shaping Degradative Logic, Receptor Cycling, and Synaptic Output

Proton micro-domains exist in the endolysosomal system regardless of the overall acidity of the system. The heterogeneity of lysosomal luminal pH is determined by the location of V-ATPases, curvature, electrostatic interactions between the lumen and cargo, and the dynamic fusion/invagination process that generates time-dependent acidification micro-zones and oscillatory patterns similar to those produced by mitochondrial metabolic oscillations; however, this occurs at different frequencies and amplitudes [112].

Narrow intermembrane clefts (<20 nm) at the ER–lysosome and mitochondria–lysosome nanojunctions allow for directional proton transfer based upon the lipid composition of the junctions. Acidic phospholipids, such as phosphatidylinositol (3.5) P_2_, can act as pH-dependent proton traps and modulate the retention of protons in the interfacing space and alter the activation window of hydrolases; therefore, proton micro-transfer can conditionally direct the degradative/trafficking fate of cargo by modulating the unfolding and processing rates of cargo [113].

In this context, “logic” refers to conditional routing and proton-dependent changes in the likelihood that cargo will be routed to degradation or recycling.

Synaptic vesicles utilize proton gradients to load neurotransmitters, and the pH trajectory of the vesicle lumen determines the loading stoichiometry, quantal size, and probability distribution of release. The notion that “vesicles have proton-cargo fingerprints” is a useful conceptual tool, but it is essential to recognize that there exists a relationship between the dynamics of the vesicle lumen pH and the kinetics of the transporters and cargo identity rather than a proven coding scheme. Upon exocytosis, vesicles release protons into the synaptic cleft, producing proton micro-bursts occurring over microsecond timescales that can modulate the microenvironment of receptors by changing the hydration structure, therefore modulating postsynaptic excitability in spatially dependent manners [8]. The receptor recycling from the plasma membrane is pH-dependent and mediated by pH-sensitive sorting: the protonation of cytoplasmic tail residues can modulate the interaction with adaptor proteins and Rabs that mediate trafficking, while the pH gradients in endosomes can determine the reaction corridor for receptor recycling and availability and regulate homeostatic plasticity through conditional routing rather than solely through synaptic weight modifications. Furthermore, pH-dependent chaperones and pH-dependent processing in the secretory pathway can modulate the folding and maturation of peptides; therefore, luminal proton architectures are necessary for the maturation of neuropeptides and the reliability of exocytic events [114,115]. Overall, lysosomes, endosomes, and vesicles can be considered as distributed proton-regulated state machines that convert micro-domain proton states into probabilistic decisions concerning degradation/recycling, vesicle maturation, and synaptic output. A summary of proton-dependent mechanisms operating in mitochondrial, endolysosomal, vesicular, cytoskeletal, and nano-junctional domains is provided in Table 2.

### 5.3. Proton–Cytoskeleton–Organelle Coupling as a Basis for Dynamic Computational Microdomains

The cytoskeleton is a pH-dependent matrix whose mechanical and electrical properties are pH-dependent. Actin filaments contain acidic residues (e.g., aspartate) whose protonation state can be modified, causing alterations to the rigidity and persistence length of the filaments and the affinity for crosslinkers. The protonation state of the acidic residues can also alter the hydration shell of the filaments and affect the binding and activity of regulatory proteins (e.g., cofilin) that modulate the mechanics of spine heads [128].

Glutamate residues at the C-terminal portion of microtubules can have their affinity for kinesin and dynein motors affected by protonation; therefore, pH gradients in the vicinity of the organelles can modulate the transport of organelles by establishing pH-dependent directional biases in the movement of cargo [129]. Proton micro-domains produced by organelles can also modulate pH gradients produced by the cytoskeleton. For instance, the high PMF of mitochondria can create “proton shadows” (reduced availability of protons in the cytoplasm) adjacent to cytoskeletal structures, while lysosomes can create proton-rich halos that modulate the spacing of microtubules and modulate microtubule-actin cross-linking, therefore creating mechanically and metabolically distinct domains [130].

Proton sensitivity of cytoskeletal elements has been correlated with reorganization of organelle positions in dendritic spines and axonal hotspots; decreased local pH decreases branching of actin filaments, and therefore the stiffness of the area, and increases the likelihood of vesicle/organelle entry, while increased local pH increases branching and restricts entry [131]. The relationships between proton gradients, mechanical states, and organelle positions can produce micro-domains in which proton activity, cytoskeletal compliance, and organelle positions together limit local signaling and metabolic capabilities. Nevertheless, the proposition that mechanical proton configurations in these micro-domains store information independently of ion current and biochemical cascades remains to be tested; although mechanical proton configurations may bias subsequent local-state transitions, the magnitude, duration, and physiological relevance of this persistence must be examined. A more conservative interpretation of micro-domains related to the tension of organelle distribution, hydration ordering, and proton density suggests that these micro-domains may persist for a sufficient period of time to direct short-term signaling pathways without necessitating protein synthesis or long-term structural reorganization [132].

## 6. Multiphysics Integration, Emergent Attractor Landscapes, and the Thermodynamic Substrate of Neural Computation

The environment of a neuron’s electrical signaling is constantly being modified by various physical forces, including the electrical field, the distribution of protons, the orientation of water molecules, the elasticity of the cytoskeleton, the shape of the cell membrane, and the transition between soft matter phases. The relationship between these different physical forces is complex and each physical domain is dependent on all the others. Small amounts of environmental disturbance—such as a slightly increased amount of locally available protons, a small electrical fluctuation, or a slight increase in the rigidity of filaments—can be transmitted throughout the cell across the many physical and chemical domains, and can produce physical configurations that last long enough to impact signal processing and signal response characteristics of the neuron [51]. Therefore, it is reasonable to hypothesize that neural computing is not solely based upon the electrical and chemical properties of synapses and ion channels, but may also be influenced by the physical and energetic properties of the neuron itself during active signaling. Specifically, we can propose that the neuron will often exist in a physical “state space” of interconnected physical properties—such as curvature, tension, hydration layer ordering, local proton activity, and the viscosity of protein-based condensates—that will evolve into metastable “attractor-like” states, which can influence subsequent physical and computational behaviors of the neuron [85]. In this chapter, “attractor-like” will refer to the concept of physical and/or energetic states of the neuron that have a high degree of reproducibility and stability, as well as sufficient duration to affect subsequent computations and/or signaling behavior of the neuron. It does not imply that the state has been completely analyzed in terms of its underlying dynamical system. Rather, the following sections describe the mechanisms by which interactions across different physical domains can result in metastable states of the cell, how these states may be organized into multi-scale “attractor landscapes,” and how thermodynamic interactions among the energetic reservoirs that sustain neural activity can contribute to both stability and plasticity of the states.

In summary, multiphysics coupling is a plausible mechanism for achieving state dependence that goes beyond classical electrophysiology, but the interpretation of attractor-like states is a hypothesis that needs to be tested using falsifiable methods.

### 6.1. Cross-Domain Interfaces as Nanometric Transducers of Electrical, Mechanical, and Protonic Signals

There are interfaces that are several nanometers wide and are capable of influencing microcircuit scale behavior through non-linear magnification of perturbations in electrical, mechanical, and protonic signals. For example, small changes in electrical potential (~3–7 mV) can cause changes in the lateral pressure profile of the plasma membrane (~20–70 mN/m) and curvature strains (<1%) in the plasma membrane that are sufficient to alter the energy landscape of PIEZO1, TRAAK, or TREK-2 channels [133]. Concomitantly, mechanical deformation can alter the position of first shell hydration water, thereby altering proton transport kinetics (~30–80 femtoseconds) through the change in hydrogen bonding and thus the proton transfer rate across membranes and nanojunctions [134].

At the interface of mitochondria and the endoplasmic reticulum (average separation of ~9–24 nm), ordered hydration layers can form proton conducting chains that have an effective conductivity significantly higher than bulk cytosol [135]. In such configurations, protons can be transferred from cristae microcompartments that have a curvature-confinement generated deviation in pH (>0.5 units) to ER channels and pumps via reorientation of hydrogen bonds instead of diffusion. Such protonic perturbations can modulate the probability of opening of the IP_3_ receptor, SERCA turnover, and MCU-MICU responsiveness to oxidative phosphorylation rhythms and Ca^2+^ microdomains. Moreover, phase separated condensates that are located within ~50–200 nm of channels can modify the local dielectric environment, buffer energetic fluctuations, and modify kinase reaction fields [136]. Furthermore, a small number of protonation state shifts in condensates can alter their viscosity by ~40% in 0.02–0.05 pH units, thereby affecting phosphorylation states and channel clustering. Taken together, these findings indicate that interfaces between domains can serve as hubs of transduction where electrical events, mechanical forces, and protonic ensembles can regulate each other in order to generate state-dependent changes in excitability and signaling capability [137].

In summary, there are experimentally verified interfaces in neurons where relatively minor changes in electrical or mechanical stimuli can lead to nonlinear changes in proton transport, curvature, and biochemical organization.

### 6.2. Multiscale Organization and Emergent Attractor States in the Neuronal Physical Space

Collective perturbations that occur at the nanoscale can lead to physical configurations at the mesoscale that persist long enough to influence the processing of signals, the responsiveness of synapses, and the computation in dendrites. At sub-micrometer scales, small shifts in the charge distribution of the cytoskeleton—such as protonation-induced relaxation of actin bundles or altered electrostatic fields surrounding microtubules—can redirect vesicular and organelle movement through alteration of the probability of attachment of dynein and kinesin [138]. At micron scales, these effects can generate stable geometric and mechanical patterns, including localized asymmetric stiffness, persistent curvature of ER tubules, and micro-regions of elevated proton availability due to mitochondrial positioning. Sufficiently long-lived to potentially act as metastable states in a coupled physical state space defined by curvature, tension, hydration-layer order, mitochondrial metabolic production, and condensate viscosity, these physical configurations can impose constraints on subsequent neuronal responses, representing recent history not as a symbolically encoded memory, but as a material state that influences future transition probabilities [139].

At even larger scales, rhythmic fluctuations in metabolic and mechanical activity—ranging from ~0.03–0.5 Hz in proton motive oscillations to ~0.1–1.2 Hz in redistribution of cytoskeletal tension and ~0.01–0.1 Hz in waves of lysosomal acidification—can couple physical states of the neuron with ongoing patterns of activity across large numbers of neurons. Through modulation of the tension of the extracellular matrix and proton buffering by astrocytes, the extracellular matrix and astrocyte processes can couple physical states of individual neurons over tens to hundreds of micrometers [140]. Such coupling can help to stabilize transitions between network states by incorporating computation within extended physical manifolds that span multiple modes and spatial scales. As a result, in this context, attractor-like states would represent not only patterns of informational inputs, but also the energetic and material configuration of the neuron’s operational environment [141].

In summary, there is a plausible model of how coupling at multiple scales can generate metastable physical states that influence subsequent dynamic behavior; however, whether these represent attractor states with computational significance is a testable proposal.

### 6.3. Thermodynamic Coupling, Energy Partitioning, and Dissipative Stabilization in Multiphysics Computation

Multiphysics computing, which couples electrical, mechanical, and protonic functions, requires thermodynamic exchange of energy among energetic reservoirs that sustain neural activity. Electrical reservoirs are metabolically expensive to maintain (cost > 10^7^ J/neuron/day) and dissipate energy through gating events that range in size from ~5 to 40 zJ. Elastic energy in mechanical reservoirs is stored in cytoskeletal lattices: spectrin networks can store ~20–80 zJ of elastic energy per periodic segment when strained, whereas actin filaments can store ~1–3 kT of elastic energy per 10 nm segment depending on cross-linker density and protonation state [7]. Protonic reservoirs add additional energetic structure to the system. Mitochondria store ~3 × 10^−13^ J per organelle as proton motive force, and proton wells at the cristae apexes may affect the conversion efficiency in ways that will propagate into mechanical and electrical stability [142].

Energetic exchange occurs through distributed dissipation pathways. Entropy and enthalpy buffers provided by phase-separated condensates can tune viscosity from ~5–20 Pa·s to >80 Pa·s under mechanical or pH stress conditions, thereby maintaining the stability of kinase networks against fluctuations in electrical stimulation. Femtosecond time-scale dissipation of electrostatic disturbances by hydration layers can prevent the buildup of uncontrolled electrical charges. Filaments of cytoskeletal elements can distribute mechanical stresses over tens of micrometers, thereby preventing localized failures and maintaining the positioning of organelles [143,144,145]. Together, these dissipation pathways can facilitate convergence to metastable configurations rather than to disordered fluctuations, and thus sustain activity-dependent transformation capacity [146].

From a thermodynamic viewpoint, computation can be described as the evolution of a coupled energetic landscape in which electrical potentials, mechanical stiffness, and protonic ensembles interact across spatial scales [147]. The neuron can therefore be viewed as a multi-reservoir, nonequilibrium system whose computational capacity depends on its ability to stabilize, modulate, and transform energetic fluctuations into structured physical states that influence information processing and response trajectories across both spatial and temporal scales [148].

## 7. Pathological Fragmentation of the Multiphysics Computational Substrate: Early Biophysical Failure Modes and Cascading Breakdown Across Scales

Most neurological pathology arises directly out of the distortion of the multiphysical architecture of neurons, which brings together the physics of electrical gradients, mechanical tension fields, proton ensembles, and soft matter phases. Most of the initial distortion occurs in structures that are smaller than what could normally be detected by biomarkers and occur between 0.5 and 50 nm within the hydration networks of the neuron, where the dielectric constant has been modified and the energy boundary is losing coherence [149]. As the distortions continue to grow, they create instability in the physical attractors of the neuron, lower the accuracy of the neuron’s energy cycles, and interfere with the neuron’s ability to transition to different states of computation. The neuron responds to these distortions systemically, so even minimal distortions to proton transfer topology, curvature elasticity or condensate viscosity will propagate upwards to produce misaligned cytoskeletal tension fields, erratic metabolic signals, and ultimately a collapse of the circuitry [150]. Unless explicitly stated otherwise, the numerical ranges below reflect values reported in experimental studies where available, and otherwise represent illustrative estimates grounded in mechanistic models or biophysical scaling arguments derived from the cited literature; where numbers are model-derived, this is indicated.

Below are descriptions of previously unknown types of pathological mechanisms that arise from failure of the multiphysical substrate of the neuron and represent the multiple dimensions of computational deterioration that occur prior to the development of overt synaptic or structural degeneration [151,152].

### 7.1. Dielectric Drift, Electromechanical Misalignment, and Instabilities of Voltage-Sensing Architectures

A fundamental component of high quality electrical computation is the very narrow dielectric landscape that describes the electric field experienced by the voltage sensing domain, determines the gating charge chemistry, and restricts the formation of nanoscopic clusters of channels. This landscape loses coherence due to slight physicochemical changes that are incapable of being recognized as classical dysfunction of ion channels when it becomes unstable, and thus pathology emerges [153]. Dielectric drift represents one of the earliest failure modes in this regard, where oxidative remodeling of polyunsaturated lipids or changes in the composition of phosphoinositides lead to changes in the dielectric properties in patches of approximately 2–5 nm surrounding the voltage sensing domain. Local dielectric changes of ΔE = 3–10 are measured in patches of 2–5 nm [154,155]. Small changes in dielectric properties will alter the displacement of the S4 helix and, correspondingly, the electric field coupling, affecting gating charge transfer by 8–18% without complete loss of the channel. Thus, the synchronization of channel openings among nanoclusters of channels is disrupted and the tight temporal correlations required for high frequency firing are lost [156].

Another type of mechanism is electro-mechanical misalignment at the plasma membrane/cortex interface. Unbalanced protonation of the anchoring site of actin and spectrin, commonly producing anomalous mechanical tension at the surface of the cortex, causes deviations of 10–30 nm in the curvature radius of the membrane, thus changing the axial tilt sensed by the voltage sensor in channels such as Nav1.6 or Cav2.1 [157,158]. Altered mechanical loading produces sub-threshold oscillations, increases the noise of gating, and produces a millisecond-scale jitter that contributes to a decrease in the precision of spike timing long before conduction blockages occur [159]. Hydration shell decoherence around channel nanodomains may be considered as a third mechanism. Water molecule orientation near voltage sensors is stabilized by normal proton transfer pathways around the channel nanodomain; however, when fluctuations in the proton gradient of the mitochondria or lysosomes occur, these pathways are disrupted. Changes in hydration shell dynamics of as little as 0.02–0.05 pH units can modify the hydrogen bonding network of water and therefore modify the relaxation times of hydration shells by 20–60 fs. The electromechanical coupling constants of voltage gated channels are affected by changes in hydration shell dynamics [160,161]. Altogether, the early distortions in the dielectric and mechanical properties of the membrane disrupt electrical homeostasis and create a fluctuating excitability landscape that is a precursor to the appearance of clear synaptic pathology [162].

### 7.2. Mechanical, Curvature, and Cytoskeletal Failure as Drivers of Subcellular Energetic Decoupling

Mechanical stability is necessary for maintaining a morphologically stable shape of the neuron, proper positioning of organelles, and appropriate spatial alignment of reaction micro-domains. However, pathological changes in the mechanical properties of the neuron, curvature, or filament structure significantly affect the fidelity of metabolic and signaling processes of the neuron, and these effects propagate upward through the neuron. Curvature fatigue is one of the earliest failure modes in this category resulting from repeated high frequency synaptic activity or metabolic overload [84,163]. High frequency synaptic activity and metabolic overload result in the exhaustion of the curvature generating apparatus. The BAR-domain proteins, dynamis spirals, and endophilin scaffolds lose curvature responsiveness when membrane tension fluctuates outside of their optimal range. Changes of 20–40 nm in the curvature radii disrupt the geometry of vesicle fission and produce vesicles with sub-optimal proton loading capacity. Vesicles with sub-optimal proton loading capacity are characterized by poor packaging of neurotransmitter, non-uniform quantal release, and distorted release probability distributions across synaptic trains [164].

A second mechanical vulnerability is the softening of the cytoskeleton. Softening of the cytoskeleton is the result of shifts in the protonation equilibrium of acidic patches located on the surfaces of actin, spectrin, and microtubules. Filament stiffness is decreased from baseline values of ~1300 pN to 700–900 pN based upon local proton concentrations and disrupts the flow of forces through the cytoskeletal lattice. Processivity of transport motor activity is decreased and pauses are increased by 20–40%. Erratic motion of organelles is produced and mitochondria fail to travel to high demand areas, creating local reductions in ATP-to-ADP ratios and micro-domain specific energy deficits that cannot be detected using bulk metabolic assays [165]. Discontinuity of the mechanical skeleton that supports the organization of sodium channels at the nodes of Ranvier due to rupture sizes of <70 nm is a third failure mode of axial mechanical decoherence. The mechanical skeleton distorts the mechanical loading applied to sodium channels, and the hotspots of conduction are no longer aligned, reducing the reliability of conduction during high frequency stimulation and contributing to the slowing of cognition that is present early in degeneration [166].

### 7.3. Protonic, Redox, and Soft-Matter Phase Instabilities as Drivers of Computational Collapse

In addition to electrical and mechanical dysfunction, many of the earliest and most severe pathologies are a result of dysfunction in the protonic and soft matter domains that regulate metabolic, degradative, and regulatory processes [167].

Protonic decoherence is a primary failure mode because of disruption of the cristae of mitochondria, alkalinization of lysosomes, or widening of ER nanojunctions. Normally, proton oscillations coordinate the workload of mitochondria with calcium microdomains and the redox state [168]. When the curvature of the cristae is flattened by 15–25 nm, proton wells dissipate, and the amplitude of oscillation of the proton motive force is reduced by 20–50%. Widening of ER–mitochondria nanojunctions from 10–25 nm to 30–45 nm breaks the hydrogen bonded proton conduction chains and decouples calcium flux from metabolic availability [169]. The coordination of energy is disrupted, and localized metabolic noise is generated that impairs synaptic integration. Redox-phase coupling failure is the second mechanism. Compromised antioxidant capacity generates irregular redox micro-domains and alters the electron transfer rate in the respiratory complex by 5–12%. Even slight redox modifications alter the efficiency of proton pumping, and stochastic fluctuations replace rhythmic proton gradients. These fluctuations propagate to condensate bound kinases and membrane bound reaction fields and reduce the fidelity of phosphorylation [61].

The third and potentially most destructive pathology is the stiffening of condensates and the vitrification of soft matter. Proteins containing intrinsically disordered regions, such as TDP-43, FUS variants, hnRNPA1, or tau, undergo phase boundary shifts as a function of protonation pattern changes or redox stress. Condensates shift from liquid-like states (viscosity 5–20 Pa·s) to gel-like or semi-solid states (50–120 Pa·s). Molecular turnover rates are reduced by an order of magnitude [170]. Such phase transitions entrap regulatory enzymes, halt mRNA traffic, disrupt translation hotspots, and alter kinase/phosphatase balances across synapses [171,172]. Once the protonic coherence is disrupted, the condensates are hardened, and the redox variability is increased, the neuron loses the physical alignment needed to stably support the attractor states. The cell enters a state where the multiphysics interactions do not converge to coherent energy minima but are instead chaotic, allowing for further damage regardless of whether there are genetic mutations or protein aggregates. Finally, these silent nanoscale disruptions result in the macroscopic manifestation of cognitive decline, synaptic failure, and degenerative disease [173,174].

## 8. Conclusions: Toward a Physics-Integrated Neurobiology of the Next Century

The physical framework that was explored throughout this paper offers a way to view the function of neurons that is different than the classical approach of separating electrical, biochemical, and metabolic processes. The evidence presented throughout this paper demonstrates that the operation of a neuron is based on a continually changing configuration of coupled physical variables—electrodynamic potentials, mechanical stress fields, proton-encoded, free-energy gradients, hydration layer ordering, phase transition thresholds, and nanoscale junction geometries—that provide the necessary conditions for the maintenance of the stability required for thought. It appears that these physical processes occur within a space where the differences between signal, structure, and metabolism are eliminated and where the output of computations are determined by the coordinated deformation of material states at multiple levels of organization. Therefore, if the previous three decades of neuroscience research were defined by the ion channel and the synapse, it appears that the next three decades of neuroscience research may be defined by the previously ignored but critical physical degrees of freedom that determine how neural tissues store, propagate, and transform information. A common theme that is evident throughout the work presented here is that physical parameters that have been considered secondary for many years (e.g., membrane curvature; dielectric heterogeneity; local proton activity; nanoscale mechanical compliance; changes in soft matter viscosities) can influence function with temporal and spatial resolution similar to that of ion flux or protein signaling. The proton micro-domains created at mitochondria–ER contact sites or at synaptic vesicles, the curvature-dependent electromechanical transduction occurring at membrane ridges, the hydration layer ordering influencing proton mobility, and the phase behaviors of the nanoscale condensates all combine to describe a single, unifying physical logic for the neuron. These physical parameters add additional computational freedoms to the cell and allow it to encode history into its mechanical posture, hydration architecture, or energy landscape curvature and thereby create types of memory that are independent of the traditional genomic and synaptic mechanisms of memory formation. This type of cross-disciplinary integration suggests that rather than functioning as an electric circuit, the neuron is better viewed as a dynamic dissipative system made up of matter that computes because of continuous coupling between energetic, mechanical, and structural order parameters.

There may also be significant implications for health and disease. In many cases of neurological disorders, including early stages of Alzheimer’s disease and metabolic neurodegeneration, we are observing disruptions not only in molecules and circuits but in the physical conditions that are required for stable computation—e.g., dysregulation of proton gradients, loss of precision in nanojunctions, loss of mechanical coherence, condensation of matter, or disruption of hydration layer ordering. These disruptions alter the physical manifolds upon which neural computation relies and therefore lower the energetic barriers that protect against degradation and permit transitions into unstable or maladaptive states. Understanding these physics-based vulnerabilities may lead to earlier diagnosis of diseases and design of treatments that restore the physical conditions necessary for neuronal resilience (i.e., proton motive force gradients, mechanical integrity, phase fluidity, and nanojunction alignment). In looking forward, the field is poised at the threshold of a much broader synthesis. The tools needed to study these domains (i.e., time-resolved cryogenic tomography, ultra-fast proton tracking spectroscopy, molecular dynamics simulations of hydration layer ordering, high resolution mechanical interferometry, and AI-driven reconstruction of multi-physics states of living cells) are only now maturing. When used in concert, they may ultimately enable studies that map in vivo the emergence of proton fluxes, forces, charges, and phases into a coherent computational landscape. Studies such as these may ultimately reveal new principles: how neurons balance energy flow and mechanical tension to stabilize attractor states; how proton currents are synchronized with enzymatic cycles to regulate plasticity; or how nanoscale phase transitions encode long range network dynamics.

The goal of this paper has been limited: to provide a conceptual foundation for the emerging discipline of physics-integrated neurobiology and to gather the existing evidence that suggests that neuronal computation is a product of the coordination of electrodynamic, mechanical, and energy-coupled physical variables. There are many unanswered questions and there is a vast expanse to the limits of this landscape that lie beyond what is currently known. However, if the concepts outlined in this paper stimulate further investigations into the physical properties of neurons (and thus encourage the examination of proton flux, curvature modulation, nanojunction geometry and phase behavior as critical components of neural function), then the goals of this paper will have been met.

Finally, we hope that the continued convergence of physics and neurobiology will continue to grow and evolve, and that this will lead to new imaginations for the field. The physical structures and energy landscapes that were discussed in this paper are merely the beginning of a much longer story—a story that has yet to be written and whose development will depend on the combined insights of future investigators. As this area continues to develop, we anticipate that new principles, new measurements, and new theoretical frameworks will arise that will deepen our understanding of the nervous system, and will reveal aspects of computation that are currently unknown. While this paper concludes, the physical biology of neural computation is far from being complete, and the issues raised in this paper may serve as mere signposts for the discoveries that await us.

## Figures and Tables

**Table 1:** Summary of the major proton-dependent mechanisms that modulate neural signaling across molecular, enzymatic, and synaptic scales, highlighting how protonation reshapes conformational states, reaction kinetics, vesicle physiology, and integrative microcircuit behavior, thereby illustrating proton flux as a rapid, low energy regulator of neuronal computation.

Domain	Core Proton-Dependent Physics	Functional Impact on Neural Computation	Representative Correlates	References
Ion-channel microstates & gating landscapes	Protonation of acidic clusters reshapes hydration shells, dielectric gradients, and S4 energy surfaces; proton-sensitive hydrogen-bond rewiring in AMPA/NMDA receptors; proton-coupled “ratcheting” in transporters stabilizes intermediate states	Shifts activation thresholds, desensitization trajectories, Ca^2+^ nanodomain geometry, and vesicle loading energetics; real-time tuning of excitability across subcellular microdomains	Time-resolved cryo-EM, ultrafast IR spectroscopy, QM/ML gating-surface simulations	[73]
PCET enzymes, phosphorylation cycles & proteome dynamics	Proton-coupled electron transfer modifies redox microdomains; protonation alters catalytic geometry in kinases/phosphatases; pH-dependent sticker–spacer cohesion shifts condensate material states; filament stiffness adjusts via proton-sensitive actin–binding interactions	Modulates phosphorylation bandwidth (MAPK, CaMKII), ROS topology, condensate permeability, local translation, and activity-dependent spine mechanics	Single-molecule kinetics, PCET spectroscopy, LLPS rheology, pH-aware MD	[74]
Synaptic microphysiology, vesicle gradients & fusion energetics	Cleft proton microbursts reorganize receptor hydration and clamshell energetics; V-ATPase proton gradients set neurotransmitter loading stoichiometry; protonation of lipid headgroups adjusts curvature stress and fusion-pore mechanics	Tunes EPSC amplitude, release probability, synchronous/asynchronous fusion mode, dendritic Ca^2+^ microdomains, and short-term plasticity	pHluorin vesicle reporters, cleft pH nano-imaging, fusion-pore assays	[75]
Membrane dielectric fields & hydration-shell electrodynamics	Protonation alters surface charge, interfacial water structure, lateral pressure profiles, and curvature fields; modifies ion mobility and polarization of membrane microdomains	Region-specific excitability tuning; modulation of EPSP propagation, Ca^2+^/K^+^ wavefront geometry, and mechanosensitive channel behavior	2D-IR hydration mapping, quantum-trained MD of water layers, membrane-tension imaging	[76]
Dendritic/axonal proton microcircuits & metabolic coupling	Proton-dependent Ca^2+^ buffering, mito-ATP flux, and ROS generation; protonation reshapes MAM geometry and cytosolic water structuring, influencing viscosity and diffusion	Controls dendritic integration windows, metabolic–electrical coupling, spike-timing precision, and early instability modes under pH drift	Dual Ca^2+^/pH imaging, mito-pH FRET sensors, electrodiffusion solvers	[77]

**Table 2:** By organizing mechanisms according to their structural scale and biophysical context, the table highlights how proton gradients, confinement effects, hydration-layer ordering, and pH-sensitive molecular interactions collectively generate structured energetic microdomains. These phenomena introduce proton-dependent logic into metabolic regulation, cargo trafficking, mechanical remodeling, and synaptic precision, illustrating how protons serve as integrative physical variables that bridge energy flow, signaling architecture, and local computational capacity.

Subsystem/Scale	Proton-Dependent Mechanisms	Biophysical Consequences	Computational/Functional Significance	References
Mitochondrial Cristae Architecture	Curvature-induced sub-cristae proton wells; nanoconfined hydrogen-bond ordering; geometric PMF amplification	Persistent proton microplateaus; restricted lateral proton mobility	Encodes workload into spatially resolved proton gradients; microdomain-specific ATP synthesis patterns	[116]
ATP Synthase Rows & Fₒ Interfaces	Hydration-layer structuring at proton entry; proton crowding influencing rotor torque; cooperative proton gating	Rotor torque variability; ATP output heterogeneity between adjacent rows	Converts proton fluctuations into analog metabolic output signals	[117]
Mitochondria–ER Contact Sites (MERCs)	Hydration-layer proton conduction; proton–lipid relays; pH-dependent SERCA/MCU tuning	Synchronized proton–Ca^2+^ oscillations; feedback between PMF and ER Ca^2+^ load	MERCs act as proton–Ca^2+^ logic gates shaping excitability	[118]
Outer Mitochondrial Membrane Nanodomains	Cardiolipin-dependent pKa shifts; proton-tuned VDAC conductance; cholesterol-modulated proton gating	Spatial heterogeneity in metabolite flux; redox-coupled microdomains	Proton-coded control of substrate timing and metabolic responsiveness	[119]
Lysosomal Proton Stores	V-ATPase positional clustering; cargo-dependent proton buffering; curvature-induced acid accumulation	Enzymatic activation microzones; acidification hotspots	Proton-coded decisions for degradation vs. recycling	[120]
Mitochondria–Lysosome Nanojunctions	Proton-selective PI(3.5)P_2_ funnels; pH-guided hydrolase tuning; Rab7-dependent proton retention	Directed proton transfer shaping lysosomal maturation	Proton-gated cross-organelle communication governing degradation load	[121]
Synaptic Vesicles	Proton–cargo fingerprints; pH-controlled loading stoichiometry; microsecond proton bursts during exocytosis	Quantal variability; cleft hydration-shell restructuring	Proton-coded tuning of release probability and postsynaptic gain	[8]
Endosomal Recycling Networks	pH-triggered adaptor recruitment; protonation-dependent Rab transitions; gradient-directed sorting	Receptor-routing fidelity; synapse-specific receptor maintenance	pH-based routing logic for homeostatic plasticity	[122]
Secretory Pathway (Golgi → Vesicles)	Proton-sensitive chaperone dynamics; pH-dependent peptide cleavage; proton-modulated cargo condensation	Quality-controlled vesicle identity; peptide maturation fidelity	Proton-encoded precision in neuromodulator production	[123]
Actin Cytoskeleton	Protonation of acidic patches altering stiffness; pH-sensitive cofilin/profilin binding; hydration-shell shifts	Local compliance gradients; activity-linked mechanical tuning	Mechanical computation modules shaping synaptic plasticity	[124]
Microtubules	C-terminal tail protonation modulating motor affinity; proton-sensitive dielectric pathways; pH-tuned MAP interactions	Motor steering bias; cytoskeletal spacing shifts	Proton-coded regulation of organelle transport trajectories	[125]
Cytoskeleton–Organelle Mechanical Coupling	Mitochondrial proton shadows; lysosomal proton halos; pH-dependent cytoskeletal softening/hardening	Mechanical load partitioning; spatially tuned metabolic microzones	Defines computational microdomains where mechanics and metabolism converge	[126]
Nanojunction-Based Proton Highways	H-bond network directional relays; hydration-mediated proton conduction; pH-dependent protein alignment	Ultrafast conduction bypassing bulk diffusion; spatial proton bias	Structured energy-flow channels for hierarchical computation	[20]
Hydration-Layer Proton Structuring	Dielectric rearrangements near curved membranes; water-network compression; lipid headgroup protonation	Altered membrane conductivity; reorganized local electrostatics	Curvature–hydration coupling as a computational variable	[6]
Proton-Driven Phase Behavior in Condensates	Protonation of LCDs modulating sticker–spacer contacts; pH-dependent condensate fluidity; interfacial tension tuning	Phase transitions controlling permeability & reaction kinetics	Proton-encoded remodeling of biochemical workspace geometry	[127]

## Data Availability

No new data were created or analyzed in this study. Data sharing is not applicable to this article.
